# Healthy lifestyles in pre-service teachers in Israel: the impact of academic institutions

**DOI:** 10.3389/fpubh.2023.1191814

**Published:** 2023-07-20

**Authors:** Ronit Ahdut-HaCohen, Paz Carmel

**Affiliations:** ^1^Department of Medical Neurobiology, Institute of Medical Research, Hadassah Medical School, The Hebrew University of Jerusalem, Jerusalem, Israel; ^2^Department of Science, David Yellin Academic College of Education, Jerusalem, Israel; ^3^Department of Adult Education, David Yellin College of Education, Jerusalem, Israel

**Keywords:** health promotion, academic institution, healthy lifestyle, healthy eating, sports, COVID-19

## Abstract

**Purpose:**

This study examines the impact of academic institutions on changes to students’ awareness and habits regarding a healthy lifestyle, specifically through nutrition and physical exercise, following the Covid-19 pandemic.

**Design and subjects:**

In May 2020, quantitative online questionnaires were completed by 266 pre-service teachers (83.5% female), aged 19–63, who were studying at an academic institution in Israel.

**Setting:**

The questionnaire, which included health-related 15 items, as well as a number of demographic questions, was distributed *via* social media, academic mailing lists, and the researchers’ colleagues.

**Methods:**

The respondents were asked to provide socio-demographic data and information regarding their health-related habits, such as smoking and exercising, at two timepoints: prior to the Covid-19 pandemic and during the first lockdown in Israel (March–May 2020).

**Analysis:**

Statistical analysis included paired *t*-tests, Wilcoxon and McNemar tests, Pearsons’s correlations, and hierarchical regressions.

**Results:**

The academic institution’s promoting of a healthy lifestyle, as perceived by students, was found to contribute to the explained variance (EPV) of their maintaining a healthy lifestyle, prior to and during the Covid-19 pandemic (*R*^2^ = 9.4%, *p* < .001and *R*^2^ = 2.4%, *p* = 0.009, respectively), beyond the respondents’ demographic characteristics. Moreover, correlations were found between the institution’s promoting of a healthy lifestyle at both timepoints. Respondents who perceived their institution as promoting a healthy lifestyle prior to the pandemic maintained healthier lifestyles than their peers; healthier lifestyles were also maintained by respondents who were unmarried, non-smokers, more educated, and watched less television. Finally, the institution’s promoting of a healthy lifestyle prior to the pandemic significantly contributed to the students’ maintaining a healthy lifestyle and healthy nutrition during the pandemic.

**Conclusion:**

The findings of this study highlight the impact of academic institutions on maintaining healthy lifestyles, even in times of crises and emergencies, thereby contributing to public health.

## Introduction

Studies have addressed the promoting of healthy lifestyles in students of higher education, with an emphasis on positive habits such as nutrition and exercise (1). The maintaining of students’ healthy lifestyles is impacted by their social environment, relationships, living conditions, and previous habits (2, 3). Novice students encounter significant lifestyle changes, especially when living away from home for the first time. Their social environment vastly changes, and they tend to eat out more often, consuming more fat, sugar, and salt-saturated foods, combined with less fruit and vegetables. Moreover, students are not always knowledgeable about positive food purchasing habits, from both a financial and health-related aspect (4, 5).

Health is a core component in a person’s well-being, with nutrition and physical exercise being key to conducting healthy lifestyles. The World Health Organization (WHO) states that healthy lifestyles refer to behavioral aspects that a person can control as a means for improving their quality of life, such as the foods that they eat and the exercise that they perform (6, 7). Enhancing healthy lifestyles is especially important in this day and age, with people tending to lead more sedentary lifestyles that lack physical exercise and that are abundant in unhealthy foods – often leading to ailments and disease in the future (8, 9). The Covid-19 pandemic increased such undesirable lifestyles, with people sitting for longer periods of time, often in front of electronic screens, due to lockdowns and other social restrictions (10, 11). In routine situations, such behaviors are the main cause of future background diseases, such as diabetes and hypertension. Yet during the Covid-19 crisis, people worldwide - including students - completely altered their lifestyles, with increased indoors sitting, decreased outdoors exercise and visit to the gym, and increased digital screen time; In other words, during this period, the sedentary lifestyle greatly increased (12, 13).

Health is an important component in a person’s life, and as such, the public should be encouraged and educated on how to lead a healthy lifestyle, with an emphasis on nutritious eating and on physical exercise. Furthermore, not maintaining a healthy lifestyle leads to the increased probability of developing background diseases, such as hypertension and diabetes. This in turn leads to heavier financial costs for both the unhealthy person and his family, and to the health system as a whole. The term *health promotion* relates to processes and norms that are implemented by various institutions and organizations for encouraging people to make healthier decisions and avoid health-risk factors. Health promotion can be defined as, “A process designed to allow people to increase control over their health and the factors affecting health… Health promotion does not focus only on individual decisions but also on a large variety of environmental and social interventions” (14). Moreover, as healthcare systems are not able to improve public health solely by treating existing illnesses, health promotion is of the utmost importance and should be achieved through proactive and preventive measures (15).

### Health promotion in academic institutions

The concept of health promotion stems from a salutogenic approach, one that emphasizes the inadequate benefits of the health system’s attempts to improve the public’s health – simply by treating those who already suffer from ailments and disease; instead, health should be perceived as an ongoing endeavor that lasts throughout a person’s lifetime. As such, a range of environmental and other health-related conditions must be improved proactively, in advance, to prevent illness and enhance healthy living among the public (15).

The Ottawa Charter for Health Promotion, signed in 1986, aimed at promoting international dialog between politicians, academics, and health workers – based on the shared desire to promote public health and well-being (14). This charter emphasized the setting-based approach, whereby healthy activities and lifestyles should be promoted within the various settings of each and every person, including their home, work, and place of education (16). Indeed, in addition to providing a formal learning setting, educational institutions can also serve as a framework for educating people about a range of other aspects, including health-related issues, through a health-promoting culture that enhances healthy lifestyles (14, 16). Specifically, the concept of a *health-promoting university* relates to academic institutions that are part of a person’s daily setting, and as such, must be included in their related health-promoting endeavors. The health-promoting university is expected to create a supporting environment for both students and faculty, and for its community as a whole (17).

In 2015, almost three decades after the signing of the Ottawa Charter for Health Promotion, the Okanagan Charter was developed by researchers, practitioners, administrators, students, and policymakers from 45 countries (18, 19). The purpose of this charter is twofold, as it defines the important role of academic institutions in promoting healthy lifestyles among students, staff, and the nearby community, while encouraging and enabling academic cooperations based on the importance of promoting health. Indeed, this charter defines a health-promoting academic institution as one that offers a suitable infrastructure for conducting educational processes that also address the field of health; moreover, they do so as an integral part of the institution’s educational, social, and moral existence.

In academic institutions, two health-promoting approaches can be observed: First, the more academic approach, *Health for All*, focuses on the teaching and studying of healthy living and lifestyles through academic courses, that are offered to both students and to the public in general. In some cases, it is even the students themselves who provide health-promoting classes to the public. Either way, such courses and education are part of the institution’s relationship with its surrounding community, as proposed by the WHO – in an attempt to strengthen relationships between academic institutions and the towns in which they are situated, while promoting the health of residents, students, and faculty (20–22).

Compared to the theoretical-academic approach presented above, the second approach, which is the focus of this study, promotes health through more practical means and for their entire population, i.e., students, lecturers, administrators, and managers. For example, by offering more nutritious options, forbidding smoking, and requiring students (and lecturers) to exercise on campus (23, 24) – as seen in a range of academic institutions in Israel. In turn, the educators who work in such institutions could also be expected to play a significant role in instilling healthy habits in their own students, and pre-service teachers may then continue to do so for their own students as future in-service ones (25).

This research paper specifically focuses on a college in Israel that applies this approach, having introduced a large range of health-promoting activities in a clear and engaging manner, while making them an integral part of the institution’s discourse. The college addressed in this study encourages healthy eating, through the food sold in the cafeteria and served at meetings and conferences. The college also encourages physical exercise, by presenting health tips, creating dedicated walking paths, putting up signs around campus that encourage walking, and placing signs by the elevators recommending the use of the stairs instead. The college discourages smoking, through few designated open-air smoking areas and no smoking signs in all other areas. A discount on hot beverages is offered to people who use a reusable cup, open lectures are held by a range of experts on health-related issues, emails are sent out offering health-related tips and advice, and posters encouraging physical exercise are placed in the administration offices next to desks and computers – and many other health-promoting actions.

### Health promotion during the Covid-19 pandemic

It is well-established that the Covid-19 pandemic drastically impacted people’s lives and well-being (26, 27). The academic student population, for example, had to adapt to new and stressful learning environments and platforms. Online distance learning meant that students no longer walked around campus with their peers, but found themselves sitting alone in front of their computer for hours on end. As their sedentary lifestyles greatly increased, their healthy ones began to diminish (12, 13). It is also important to note that in addition to the development of *learning habits* related to knowledge acquisition and contemplation, the studying process also entails the development of *behavioral habits*, such as eating and performing physical exercise (1). Such habits are the product of the students’ social environment and interpersonal relationships, their living conditions, and even their previous eating and physical exercise habits. Moreover, studies show that during lockdown, people tended to eat more, move less, and exercise less, thereby adopting less healthy lifestyles (2, 3, 26, 27).

In line with the literature review presented above, it is clear that leading a healthy lifestyle is beneficial for both individuals and for society as a whole. Moreover, academic institutions tend to encourage healthy lifestyles. Yet the question is, do their efforts actually impact their students and their lifestyles? This is especially important to examine in pre-service teachers, as they too could play an influential role in their future students’ lives, and as such, may have a positive impact on their healthy lifestyles as well. The aim of this study, therefore, is to examine the impact of the academic institution on its students’ health-related lifestyles, specifically through nutrition and exercise. The study was conducted during the Covid-19 pandemic, yet was planned prior to this crisis. The respondents were therefore asked about their healthy lifestyles prior to and during the pandemic, enabling an important comparison. The findings of this study could contribute to the literature, providing important theoretical and practical information regarding the impact of colleges and universities on its students’ health-related behaviors, in times of routine and in times of emergencies and crises.

## Methods

### Respondents

In May 2020, between the first and second lockdown in Israel, quantitative online questionnaires were completed by 307 pre-service teachers from a teacher’s training college in Israel (86.6% response rate), of whom 266 met the inclusion criteria, having answered all of the questions and currently studying at the relevant academic institution. The 266 respondents (222 female), aged 19–63 (*M* = 33.44, *SD* ± 10.52), were studying to become kindergarten teachers, elementary school teachers, or secondary school teachers. They were asked about their education, marital status and children, smoking habits, place of residence prior to and during the pandemic, and place of work prior to and during the pandemic (Table 1).

**Table 1:** Demographic characteristics.

	Values	Frequency (%)
Gender	Male	44 (16.5%)
	Female	222 (83.5%)
Academic studies	BEd/certificate	230 (86.5%)
	MEd	36 (13.5%)
Marital status	Single	118 (44.4%)
	Married	148 (55.6%)
Children	Without children	130 (48.9%)
	With children	136 (51.1%)
Smoking	Does not smoke	219 (82.3%)
	Smokes	47 (17.7%)
Place of residence before Covid-19	With family	166 (62.4%)
	Alone or with partner	47 (17.7%)
	With parents	53 (19.9%)
Place of residence during Covid-19	Did not change place	234 (88.0%)
	Moved back to parents	32 (12.0%)
Work status before Covid-19	Did not work	34 (12.8%)
	Worked	232 (87.2)
Work status during Covid-19	Did not work	148 (55.6%)
	Worked	118 (44.4%)

Most respondents were studying for their BEd and/or teaching certificate, 1–5 days a week (*M* = 2.64, *SD* ± 1.41); approximately half were married, with 1–7 children (*M* = 2.79, *SD* ± 1.42). Most did not smoke, had been working prior to the Covid-19 pandemic, and had not changed their place of residence since the pandemic. About 60% reported having lived with their families prior to the pandemic. The number of students who were working during the pandemic dropped by about half compared to prior to the pandemic (87.2 and 44.4%, respectively).

### Tools

First, the respondents were asked to provide demographic background, including age, field of study, and smoking habits. To examine the influence of the quarantine on sports and eating habits, a 15-item questionnaire was employed during the first Covid-19 lockdown in Israel (March–May 2020), including questions on background demographics, sports, and eating habits. The data provided by the respondents was both retrospective (regarding the pre-pandemic era) and current (during the pandemic). Prior to sending the questionnaire to the potential respondents, it was validated by eight experts in the field of health promotion (28). The respondents were asked to: (1) rate seven items on a Likert-like scale of 1 (not at all) to 5 (to a great extent), such as “To what extent did the academic institution promote a healthy lifestyle before Covid-19?”; (2) answer five yes/no questions, such as “Did you engage in sports activities that made you sweat before Covid-19?”; and (3) choose the most relevant frequency of three items (never, 1–3 days a week, 4–6 days a week, or every day), such as “How often did you eat fried foods (such as chips) during Covid-19?”

### Procedure

Notices requesting the voluntary participation of pre-service teachers were published on relevant social media platforms, emails were sent out by the college’s teacher training departments via their mailing lists, and our colleagues from the college also asked their students to participate. The study was approved by the Ethics Committee of the authors’ affiliated institution and the respondents submitted their informed consent at the onset of the questionnaire.

### Statistical analysis

To examine the gathered data, statistical analysis was conducted using the SPSS software, v.26, and included paired t-tests, Wilcoxon and McNemar tests, Pearsons’s correlations, and hierarchical regressions.

## Findings

The aim of this quantitative study was to investigate the perceived health-promoting acts of a teacher’s training college in Israel and its impact of the healthy lifestyles of pre-service teachers, prior to and during the Covid-19 crisis. The results of the study are presented in this chapter.

### Changes in nutrition and physical exercise in pre-service teachers

**Figure 1:**
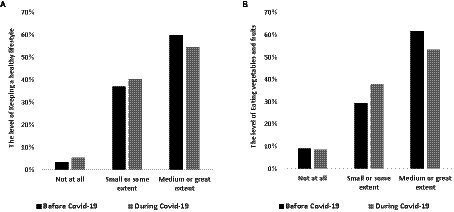
The level of keeping a healthy lifestyle and eating vegetables and fruits before and during Covid-19.

When asked to rate seven items about the college’s health-promoting acts prior to the pandemic, 40–55% of the respondents indicated that their academic institution promoted a healthy lifestyle, and provided both students and lecturers with access to information about such lifestyles, on campus and online - to a medium-to-great extent (3, 4, or 5 on the 1–5 rating scale), as seen in Table 2. Regarding nutrition, while approximately 60% of the respondents indicated their maintaining a healthy lifestyle (including eating fruit and vegetables) to a medium-to-great extent prior to the pandemic, this number decreased to about 50% during the crisis. Moreover, Wilcoxon tests showed significant differences in the extent to which the respondents maintained healthy lifestyles and ate fruit and vegetables between prior to and during the pandemic (*Z* = 2.72, *p* = 0.007; and *Z* = 2.27, *p* = 0.023, respectively]. Finally, paired sample t-tests showed that levels of maintaining healthy lifestyles and nutrition prior to the Covid-19 pandemic (*M* = 2.72, *SD* = 1.05) were significantly higher than during the pandemic (*M* = 2.55, *SD* = 1.15), [*t*_(265)_ = 2.97, *p* = 0.003], as seen in Figure 1.

**Table 2:** The institution’s promoting and the students’ awareness of Healthy lifestyles.

Items	Values	Frequency (%)
To what extent did the academic institution promote a healthy lifestyle before Covid-19?	1 (Not at all)	46 (17.3%)
	2	75 (28.2%)
	3	74 (27.8%)
	4	52 (19.5%)
	5 (To a great extent)	19 (7.1%)
To what extent did your academic institution provide students and faculty members with access to on-campus healthy lifestyle knowledge before Covid-19?	1 (Not at all)	69 (25.9%)
	2	85 (32.0%)
	3	67 (25.2%)
	4	29 (10.9%)
	5 (To a great extent)	16 (6.0%)
To what extent did your academic institution provide students and faculty members with access to online healthy lifestyle knowledge before Covid-19?	1 (Not at all)	81 (30.5%)
	2	75 (28.2%)
	3	60 (22.6%)
	4	35 (13.2%)
	5 (To a great extent)	15 (5.6%)
To what extent did you maintain a healthy lifestyle before Covid-19?	1 (Not at all)	9 (3.4%)
	2	26 (9.8%)
	3	72 (27.1%)
	4	83 (31.2%)
	5 (To a great extent)	76 (28.6%)
To what extent did you eat fruit and vegetables on a daily basis before Covid-19?	1 (Not at all)	24 (9.0%)
	2	29 (10.9%)
	3	49 (18.4%)
	4	61 (22.9%)
	5 (To a great extent)	103 (38.7%)
To what extent did you maintain a healthy lifestyle during Covid-19?	1 (Not at all)	14 (5.3%)
	2	50 (18.8%)
	3	57 (21.4%)
	4	66 (24.8%)
	5 (To a great extent)	79 (29.7%)
To what extent did you eat vegetables and fruits you eat daily during Covid-19?	1 (Not at all)	23 (8.6%)
	2	40 (15.0%)
	3	61 (22.9%)
	4	51 (19.2%)
	5 (To a great extent)	91 (34.2%)
Did you engage in sports that made you sweat before Covid-19?	Yes	131 (49.2%)
	No	135 (50.8%)
Did you engage in sports such as yoga or Pilates before Covid-19?	Yes	188 (70.7%)
	No	78 (29.3%)
Did you exercise in a gym before Covid-19?	Yes	192 (72.2%)
	No	74 (27.8%)
Did you engage in sports that made you sweat during Covid-19?	Yes	129 (48.5%)
	No	137 (51.5%)
Did you engage in sports such as yoga or Pilates during Covid-19?	Yes	181 (68.0%)
	No	85 (32.0%)
How often did you eat fried foods (such as chips) during covid-19?	Never	7 (2.6%)
	1–3 days a week	6 (2.3%)
	4–6 days a week	191 (71.8%)
	Every day	62 (23.3%)
How often did you eat savory snacks during Covid-19?	Never	10 (3.8%)
	1–3 days a week	17 (6.4%)
	4–6 days a week	157 (59.9%)
	Every day	82 (30.8%)
How often did you eat cakes and sweet snacks during Covid-19?	Never	66 (24.8%)
	1–3 days a week	45 (16.9%)
	4–6 days a week	136 (51.1%)
	Every day	19 (7.1%)

Regarding awareness of the importance of physical exercise before and during the pandemic, approximately 50% of the respondents (medium-to-great extent) stated that they engaged in sports activities that made them sweat at both timepoints; approximately 30% reported that they engaged in sports activities such as yoga and Pilates at both timepoints. McNemar tests found no significant differences between the two timepoints in the respondents’ engagement in sports activities that made them sweat or in sports activities such as yoga or Pilates, [*ᵪ^2^* = 0.01, *p* = 0.919; and *ᵪ^2^* = 0.27, *p* = 0.483, respectively]. As gyms across Israel were closed during the lockdown, the respondents were only asked about exercising in a gym prior to the pandemic; Approximately 30% replied that they had exercised in a gym. Finally, regarding unhealthy foods during Covid-19, most respondents stated that they ate fried foods (95.1%), savory snacks (90.7%), and sweet snacks (58.2%) at least 4 days a week.

### The college’s contribution to the explained level of student awareness of healthy lifestyles

After examining changes to the respondents’ nutrition and physical exercise during the pandemic, Pearson’s correlations were performed between the college’s promoting healthy of lifestyles before Covid-19 and the respondents’ awareness of healthy nutrition and exercising (Table 3).

**Table 3:** Pearson’s correlations between students’ awareness in T1 And T2, and the institution’s promoting of healthy styles in T1 (*N* = 266, *De Facto* = 264).

Students’ awareness of the importance of PE and healthy nutrition	The institution’s promoting of healthy lifestyles
Engaging in PE before Covid-19	0.01
Maintaining healthy lifestyles and nutrition before Covid-19	0.34***
Engaging in PE during Covid-19	0.06
Maintaining healthy lifestyles and nutrition during Covid-19	0.19**
Avoiding snacks and unhealthy foods	0.08

**p < 0.01, ***p < 0.001.

Our findings indicate a positive relationship between the college’s promoting of healthy lifestyles prior to the pandemic and the respondents’ maintaining of a healthy lifestyle, including nutritional eating, before and during Covid-19 [*r*_(264)_ = 0.34, *p* < 0.001; and *r*(264) = 0.19, *p* = 0.002, respectively]. No significant correlations were found between the institution’s promoting of healthy lifestyles before Covid-19 and the respondents’ engaging in physical exercise before and during Covid-19 – and the respondents’ avoidance of snacks and unhealthy foods at both timepoints.

### The college’s contribution to awareness of healthy lifestyles prior to the pandemic

After examining the respondents’ physical exercise and nutrition prior to Covid-19, we examined the college’s promoting of healthy lifestyles and its contribution to the respondents’ awareness of healthy lifestyles at this timepoint. Hierarchical regression analysis was conducted (Table 4) to assess the college’s contribution to the EPV of the respondents’ engagement in physical exercise and their maintaining healthy lifestyles and nutrition prior to the pandemic, beyond the background characteristics, which were entered stepwise (i.e., only characteristics that significantly contributed to the EPV were entered, and in order of their significance). Next, the college’s promoting of healthy lifestyles prior to the pandemic was also entered stepwise, with this variable only being entered into the regression model if it significantly contributed to the EPV of engaging in physical exercise and maintaining healthy lifestyles and nutrition, beyond the background characteristics.

**Table 4:** Hierarchical regressions for awareness of healthy lifestyles in T1 by background characteristics and the institution’s promoting of healthy lifestyles in T1.

Steps	Explanatory variables	*B*	Standard error of B (SEB)	*β*	*R* ^2^	*∆R* ^2^
*PE in T1*						
1	Marital status^1^	−0.44	0.14	−0.20***	0.039***	0.039***
	Marital status^1^	−0.49	0.13	−0.22***		
	Gender^2^	−0.53	0.18	−0.18**	0.070***	0.031**
	Marital status^1^	−0.52	0.14	−0.24***		
	Gender^2^	−0.59	0.18	−0.20***		
	Education^3^	0.38	0.19	0.12*	0.084***	0.014*
*Healthy lifestyles in T1*						
1	Number of daily hours watching television	−0.10	0.03	−0.18**	0.033**	0.033**
	Number of daily hours watching television	−0.09	0.03	−0.16**		
	Smoking^4^	−0.40	0.17	−0.15*	0.053***	0.021*
	Number of daily hours watching television	−0.09	0.03	−0.17**		
	Smoking^4^	−0.40	0.17	−0.15*		
	Age	0.01	0.01	0.12*	0.069***	0.016*
2	Number of daily hours watching television	−0.08	0.03	−0.15*		
	Smoking^4^	−0.37	0.16	−0.14*		
	Age	0.01	0.01	0.11*		
	Level of the institution’s promoting of healthy lifestyles before Covid-19	0.31	0.06	0.31***	0.163***	0.094***

*p < 0.05, **p < 0.01, ***p < 0.001.

1 Marital status: 0 = Not married, 1 = Married.

2 Gender: 0 = Males, 1 = Females.

3 Education: 0 = BEd or certificate, 1 = MEd.

4 Smoking: 0 = Non-Smoker, 1 = Smoker.

Correlation analysis indicates that the institution’s promoting of healthy lifestyles prior to the pandemic significantly contributed to the EPV of the students’ maintaining healthy lifestyles and nutrition at that timepoint, beyond the background characteristics (*R*^2^ = 9.4%, *p* < 0.001). Moreover, the positive β coefficient shows that respondents who perceived the college as promoting healthy lifestyles prior to the pandemic maintained healthier lifestyles and nutrition at that timepoint.

Regarding the contribution of background characteristics, marital status, gender, and level of education were found to significantly contribute to the respondents’ EPV of engaging in physical exercise prior to the Covid-19 pandemic (*R*^2^ = 8.4%, *p* < 0.001), with negative β coefficients for marital status and gender, and positive β coefficients for education. More specifically, unmarried male respondents with greater education had engaged in physical exercise to a greater degree prior to Covid-19. In addition, the number of hours spent watching television prior to the pandemic, smoking habits, and age significantly contributed to the EPV of the respondents’ maintaining healthy lifestyles and nutrition prior to Covid-19, with negative β coefficients for the number of hours spent watching television and smoking habits, and positive β coefficients for age. As such, respondents who watched television for fewer hours and did not smoke prior to covid-19 were found to have also maintained healthier lifestyles and nutrition prior to the pandemic.

### The college’s promoting of healthy lifestyles prior to the pandemic and the explained levels of respondents’ awareness of healthy nutrition and physical exercise during the pandemic

We then examined the contribution of the college’s promoting of healthy lifestyles prior to Covid-19 to the EPV of the respondents’ physical exercise, maintaining healthy lifestyles and nutrition, and avoiding snacks and unhealthy foods during the pandemic, beyond background characteristics. Hierarchical regression analysis was conducted (Table 5). First, the background characteristics were entered stepwise. Next, the level of the institution’s promoting of healthy lifestyles before Covid-19 were entered stepwise, as were the students’ levels of physical exercise and maintaining healthy lifestyles and nutrition during Covid-19. Hierarchical regressions were also conducted for the level of the respondent’s awareness of the importance of physical exercise and healthy nutrition during Covid-19 by their background characteristics, the institution’s promoting of healthy lifestyles, and the respondents’ awareness of the importance of physical exercise and healthy nutrition prior to the pandemic.

**Table 5:** Hierarchical regressions.

Steps	Explanatory variables	*B*	SEB	*β*	*R* ^2^	*∆R* ^2^
*Engaging in PE during Covid-19*						
1	Children^1^	−0.29	0.10	−0.17**	0.029**	0.029**
2	Children^1^	−0.23	0.10	−0.14*		
	Preforming PE before Covid-19	0.20	0.05	0.27***	0.100***	0.071***
*Maintaining healthy lifestyles and nutrition during Covid-19*						
1	Number of daily hours watching television during Covid-19	−0.09	0.02	−0.23	0.051***	0.051***
	Number of daily hours watching television during Covid-19	−0.09	0.02	−0.21		
	Change in place of residence during Covid-19^2^	0.48	0.21	0.14	0.070***	0.018*
	Number of daily hours watching television during Covid-19	−0.08	0.02	−0.19		
	Change in place of residence during Covid-19^2^	0.49	0.21	0.14		
	Smoking^3^	−0.39	0.18	−0.13	0.086***	0.016*
2	Number of daily hours watching television during Covid-19	−0.07	0.02	−0.18		
	Change in place of residence during Covid-19^2^	0.49	0.21	0.14		
	Smoking^3^	−0.37	0.18	−0.12		
	Level of the institution’s promoting of healthy lifestyles	0.17	0.07	0.16	0.110***	0.024**
	Number of daily hours watching television during Covid-19	−0.04	0.02	−0.09		
	Change in place of residence during Covid-19^2^	0.37	0.16	0.10		
	Smoking^3^	−0.11	0.14	−0.04		
	The institution’s promoting of healthy lifestyles	−0.04	0.05	−0.04		
	Maintaining healthy lifestyles and nutrition before Covid-19	0.70	0.06	0.64	0.453***	0.343***
	Number of daily hours watching television during Covid-19	−0.04	0.02	−0.09		
	Change in place of residence during Covid-19^2^	0.36	0.16	0.10		
	Smoking^3^	−0.12	0.14	−0.04		
	The institution’s promoting of healthy lifestyles	−0.03	0.05	−0.03		
	Maintaining healthy lifestyles and nutrition before Covid-19	0.67	0.06	0.61		
	Engaging in PE before Covid-19	0.10	0.05	0.10	0.461***	0.009*
*Avoiding snacks and unhealthy foods during Covid-19*						
1	Number of daily hours watching television during Covid-19	−0.03	0.01	−0.15*	0.023*	0.023*
	Number of daily hours watching television during Covid-19	−0.03	0.01	−0.15*		
	Age	0.01	0.00	0.13*	0.041**	0.017*
	Number of daily hours watching television during Covid-19	−0.03	0.01	−0.15*		
	Age	0.01	0.00	0.26**		
	Children^1^	−0.19	0.08	−0.19*	0.062***	0.021*
	Number of daily hours watching television during Covid-19	−0.02	0.01	−0.11*		
	Age	0.01	0.00	0.21**		
	Children^1^	−0.16	0.08	−0.16*		
	Maintaining healthy lifestyles and nutrition before Covid-19	0.12	0.03	0.25***	0.121***	0.059***

*p < 0.05, **p < 0.01, ***p < 0.001.

1 children: 0 = Does not have children, 1 = has children.

2 Change in place of residence during Covid-19: 0 = Did not change, 1 = Moved back in with parents.

3 Smoking: 0 = Non-Smoker, 1 = Smoker.

Our findings indicate that the college’s promoting of healthy lifestyles prior to the pandemic significantly contributed to the EPV of the respondents maintaining healthy lifestyles and nutrition during the pandemic, beyond their background characteristics (*R^2^* = 2.4%, *p* = 0.009). The positive β coefficient indicates that pre-service teachers who perceived their academic institution as promoting healthy lifestyles before Covid-19 maintained healthy lifestyles and nutrition during Covid-19. In addition, the engaging in physical exercise before Covid-19 significantly contributed to the EPV of the respondents’ engaging in physical exercise and maintaining healthy lifestyles and nutrition during Covid-19 – with positive β coefficients. Moreover, the level of maintaining healthy lifestyles and nutrition before Covid-19 significantly contributed to the EPV of maintaining healthy lifestyles and nutrition and of avoiding snacks and unhealthy foods during Covid-19 – also with positive β coefficients.

Regarding the contribution of the background characteristics, a significant contribution was seen for the respondents’ having/not having children status to the EPV of their engaging in physical exercise during the pandemic. The negative β coefficient indicates that respondents without children tended to engage more in sports during Covid-19 than their counterparts with children. Certain background variables (i.e., number of hours watching television during Covid-19, change in the place of residence during Covid-19, and smoking habits) significantly contributed to the EPV of the respondents’ maintaining healthy lifestyles and nutrition during the pandemic. Specifically, negative β coefficients were seen for the number of hours watching television and smoking habits, while a positive β coefficient was seen for place of residence. In other words, the respondents who watched TV for fewer hours, did not smoke prior to Covid-19, and were older – tended to maintain healthier lifestyles and nutrition during the pandemic. Finally, a significant contribution was seen for three background variables (number of hours watching television during Covid-19, age, and having/not having children) to the EPV of avoiding snacks and unhealthy foods during the pandemic, whereby negative β coefficients were seen for the number of hours watching television and the children status, while positive β coefficients were seen for age.

## Discussion

This study examined the impact of an academic institution’s health-promoting acts on the healthy lifestyles of pre-service teachers, prior to and during the Covid-19 pandemic, based on nutrition and physical exercise. Our results indicate that healthy lifestyles were maintained to a lesser degree during the pandemic, compared to the period prior to the Covid-19 outbreak. For example, respondents reported an increased tendency to eat non-healthy foods, such as sweet and savory snacks and fried foods. No correlation was seen with regards to physical exercise, except that they reported having engaged in sports activities that made them sweat to a slightly greater degree prior to Covid-19. These findings are in line with other related studies that show that in times of lockdown, isolation or quarantine, people tend to exercise less and eat less healthy foods (29, 30). As such, even if the college did continue to encourage healthy lifestyles, this was much harder to do under such unique and unexpected circumstances. Moreover, the decreased certainty and increased anxiety and stress due to the pandemic led to the overeating of unhealthy foods. This has especially been seen among students who no longer left their homes but sat in front of screens for most of the day, barely moving, overeating, and not investing the time or money in preparing healthy meals for themselves (12, 13, 26, 27).

The findings of this study also indicates a correlation between the level of promoting a healthy lifestyle by the academic institution and healthy eating habits at both timepoints examined in this study, except for avoiding snacks. Yet no correlation was seen between the former and exercising habits. Respondents who perceived the college as significantly promoting a healthy lifestyle tended to also maintain healthy eating and lifestyles prior to the Covid-19 pandemic. Healthy lifestyles were maintain more by respondents who were not married, non-smokers, watched less hours of television, and had greater education before Covid-19. Our results also show that maintaining healthy lifestyles and nutrition before the pandemic was significantly higher than during Covid-19, when more respondents reported eating fried foods, and sweet and savory snacks. Interestingly, during the pandemic, a slight increase was reported in the engaging in sports activities such as yoga or Pilates, compared to the slight decrease that was reported in the engaging in sports that made them sweat. The reason could stem from the gyms being closed due to lockdown, and being extremely limited in use due to social restrictions even when they were open to the public – unlike yoga or Pilates that could be conducted from home via Zoom or other online platforms.

Respondents who perceived their academic institution as promoting healthy lifestyles indicated maintaining healthier lifestyles and nutrition prior to the pandemic than those who did not. Moreover, prior to Covid-19, healthier lifestyles were maintained by respondents who were not married, did not smoke, watched less television, and were more educated. The level of promoting healthy lifestyles by the institution prior to the pandemic was found to significantly contribute to students’ maintaining healthy lifestyles and nutrition during that time. Moreover, respondents who maintained healthier lifestyles prior to the pandemic also tended to do so during Covid-19. Finally, during the pandemic, unhealthy foods were usually avoided by respondents who watched less hours of television and had no children.

Academic institutions can have a positive impact on the lives of the people who inhabit it, as well as on the community around it. As shown in this research, the college that was examined in this study could be defined as a health-promoting one, since it raises the awareness of its students (and staff) regarding the benefits of performing sports and adopting healthy eating habits – especially during the times its students were studying from home and needed it the most (17, 19, 23).

### Limitations

This study offers important contributions to the theoretical and practical research literature. However, a number of research limitations should be addressed. First, the respondents in the study are all pre-service teachers from a teacher training college in Israel. As such, generalizations should be made with caution; future research could benefit from including additional academic institutions and students from different fields of study. Furthermore, future studies should continue to validate the questionnaire that was applied in this research, as it had not previously been used. However, it is important to note that the questionnaire was assessed by eight different experts from the field of health promotion, and changes and adaptations as per their feedback were implemented prior to its distribution.

As the Covid-19 outbreak took everyone by surprise, we did not collect data about the respondents’ relevant habits prior to the pandemic. Instead, the respondents were asked about their current habits and about their pre-Covid-19 habits at the same timepoint – during the pandemic, after the first lockdown in Israel. As such, this research is not longitudinal. Further studies could benefit from examining students’ healthy lifestyles upon embarking on their academic studies and then again upon completion of their first or final year of studies, for example (i.e., a longitudinal study).

Finally, the institution’s promoting of healthy lifestyles was measured subjectively *via* the respondents’ replies in the questionnaires that they submitted (i.e., self-reporting), as were their own habits and lifestyles. While self-reporting has been found to be a valid tool for conducting research (31, 32), future research could benefit from collecting data in a more objective manner.

## Conclusion

In this study, the participating college was found to have promoted healthy lifestyles prior to the Covid-19 pandemic and even during it. Our findings also indicate that when students become familiar with health-promoting lifestyles, they may incorporate this knowledge in their daily lives, even in times of crises, uncertainty, and social distancing. The findings of this study show that impact of academic institutions have a substantive influence on maintaining healthy lifestyles, even in times of crises and emergencies, thereby contributing to public health.

This study also shows how important habits are to human beings. Those with less commitments, like children and family, and those who smoke less and used to lead healthier lifestyles before covid-19 tended to maintain such lifestyles, except for eating snacks between classes when studying at home. Our findings also indicate that academic institutions play an important role in helping students live healthier lifestyles.

To the best of our knowledge, this study is the first to address the impact of academic institutions who promote healthy lifestyles (through a range of means such as tips and signs) on the eating and sports habits of pre-service teachers. Our findings highlight the importance of behavioral habits in humans, as seen in respondents who had led healthy lifestyles prior to the pandemic, and in turn, tended to continue to do so during the pandemic, despite distance learning and other restrictions, even during the lockdown. It is therefore important that colleges and universities worldwide place an emphasis on promoting healthy lifestyles among their students – in addition to providing academic education.

## Data availability statement

The original contributions presented in the study are included in the article/supplementary material, further inquiries can be directed to the corresponding authors.

## Ethics statement

The studies involving human participants were reviewed and approved by David Yellin College of Education Ethics Committee. The patients/participants provided their written informed consent to participate in this study.

## Author contributions

All authors listed have made a substantial, direct, and intellectual contribution to the work and approved it for publication.

## Conflict of interest

The authors declare that the research was conducted in the absence of any commercial or financial relationships that could be construed as a potential conflict of interest.

## Publisher’s note

All claims expressed in this article are solely those of the authors and do not necessarily represent those of their affiliated organizations, or those of the publisher, the editors and the reviewers. Any product that may be evaluated in this article, or claim that may be made by its manufacturer, is not guaranteed or endorsed by the publisher.
